# Clinical Reflections of a Nurse Orientation Programme From an Andragogical Perspective: A Descriptive Phenomenological Study

**DOI:** 10.1002/nop2.70732

**Published:** 2026-08-13

**Authors:** Semih Çayak, Sibel Öztürk Demir, Cansu Çavuşoğlu

**Affiliations:** ^1^ Department of Educational Sciences, Atatürk Faculty of Education Marmara University Istanbul Türkiye; ^2^ Department of Educational Sciences, Institute of Educational Sciences Marmara University Istanbul Türkiye; ^3^ Group Florence Nightingale Hospitals Istanbul Türkiye

**Keywords:** andragogy, clinical practice, nurse orientation, patient safety, phenomenology

## Abstract

**Aim:**

To explore how a nurse orientation programme is reflected in nurses' clinical experiences and to discuss the findings in relation to Knowles' andragogy.

**Design:**

A qualitative descriptive phenomenological study.

**Methods:**

Thirteen nurses from a private hospital in Istanbul were recruited using criterion and maximum variation sampling. Eligible nurses had completed the institutional orientation programme within the previous 3 months and had started clinical duty. Face‐to‐face semi‐structured interviews lasting 25–35 min were analysed using Colaizzi's seven‐step method. Saturation was reached around the tenth interview and confirmed through three further interviews. Rigour was supported through independent coding, researcher consensus, reflexive notes, participant validation and analytic documentation. After inductive analysis, the findings were discussed in relation to Knowles' andragogy, with particular attention to adult learning and transition support.

**Results:**

Seven themes emerged: (1) General contributions to orientation, (2) Clinical problem‐solving, (3) Theoretical‐to‐clinical transfer, (4) Simulation‐based readiness, (5) Medication administration safety, (6) Professional interaction and psychological safety, and (7) Programme improvement needs. Participants perceived orientation as reducing uncertainty, supporting organisational integration, strengthening medication safety‐related confidence, improving perceived readiness for emergencies and facilitating collegial interaction.

**Conclusion:**

Participants' accounts suggest that nurse orientation programmes may support perceived organisational integration, clinical problem‐solving and safety‐related preparedness and confidence during the transition into clinical practice. The andragogical perspective adds value by facilitating discussion of the findings in relation to adult learning principles, including relevance, prior experience, problem‐centred learning, motivation and professional autonomy.

**Implications for Nursing Practice:**

Orientation may be viewed as an ongoing support structure for nurses' perceived clinical adaptation, safety‐related preparedness and professional confidence. The findings may inform nurse educators, clinical leaders and healthcare institutions involved in designing and refining transition‐support programmes. Further multi‐source and outcome‐based research is needed to examine whether these perceived benefits correspond to observable clinical outcomes.

**Reporting Method:**

The COREQ checklist was used.

**Patient or Public Contribution:**

No patient or public contribution.

## 1 Introduction

Rapid changes associated with globalisation, technological innovation and social dynamics are reshaping required skill sets and necessitating the continuous development of workforce capacity and professional competencies (ILO 2023; WEF 2025). In this context, the International Council of Nurses identifies equipping nurses with competencies that respond to evolving health needs and expanding access to high‐quality continuing professional development as global priorities (ICN 2024). Orientation and adaptation programmes are therefore not only introductory activities but also early transition‐to‐practice support mechanisms for newly hired nurses. Healthcare institutions design professional development programmes to meet nurses' professional needs. These programmes, frequently described in the international literature as transition‐to‐practice or nurse residency programmes (Koh et al. 2023; Miller et al. 2023; Tawash et al. 2024), typically begin with structured orientation processes and provide holistic support during the transition period. Previous literature indicates that the integration of newly hired nurses into clinical practice is a multidimensional and often challenging transition process shaped by workplace support, leadership, induction programmes and the complexity of the clinical environment (Tawash et al. 2024). In clinical environments requiring high‐risk management and advanced competence, such as acute care settings, orientation training is reported to be critical for supporting patient safety and maintaining high standards of nursing care (Rauta et al. 2025).

## 2 Background

Studies on hospital‐based adaptation and orientation programmes show that programme content and implementation are closely associated with nurses' professional competence, self‐confidence and job satisfaction (Ernawaty et al. 2024; Kim et al. 2025; Liu et al. 2025). However, instability in the nursing workforce persists globally, with turnover among newly hired nurses concentrated in the early stages of the profession and first‐year turnover rates ranging from 12% to 25% (Bae 2023). The orientation and adaptation processes of experienced nurses are also often overlooked, despite the need for greater support and investment (Smith‐Miller et al. 2023). Although newly graduated and experienced nurses may both be newly hired, their learning needs may differ from an andragogical perspective. Adult learning theory suggests that prior experience shapes learning and that learning should be problem‐centred (Knowles et al. 2020). From this perspective, orientation training may support nurses' perceived preparedness, professional confidence and safety‐related awareness during the transition into clinical practice.

Existing qualitative literature has explored several aspects of nurses' orientation and transition‐to‐practice experiences, although this body of work remains uneven in scope and focus. For example, Pasila et al. (2017) systematically reviewed qualitative studies on newly graduated nurses' orientation experiences and showed that existing studies have mainly focused on orientation arrangements, the role of the preceptor, role transition and suggestions for improving orientation processes. Regan et al. (2017), in a descriptive qualitative study conducted across Canadian healthcare settings, examined transition‐to‐practice experiences from the perspectives of new graduate nurses and nurse leaders, highlighting the importance of formal orientation programmes, supportive mentors, constructive feedback and unit culture. More recently, Rae et al. (2025) examined registered nurses' experiences of a graduate nurse residency programme in Singapore and described such programmes as easing the shift from student to registered nurse and facilitating the transition to becoming a competent nurse. Taken together, these studies show that orientation and transition‐support programmes have been examined qualitatively, particularly in relation to new graduate nurses, preceptorship, organisational adaptation and role transition. However, fewer studies have examined how the components of an institutional nurse orientation programme are reflected in nurses' everyday clinical experiences after programme completion, particularly through a descriptive phenomenological approach and from an adult learning perspective. Quantitative studies report outcomes such as satisfaction, self‐confidence and perceived competence (Mohamed and Al‐Hmaimat 2024; Raqueno et al. 2025), but they do not fully explain how nurses perceive the transfer of acquired knowledge to complex clinical settings or how they make sense of this transition in clinical practice. Therefore, the perceived clinical reflections of orientation training require deeper qualitative exploration.

In this study, Malcolm Knowles' andragogy provides a theoretical perspective for discussing the findings in relation to adult learning. The framework conceptualises adult learning through six principles: ‘the learner's need to know’, ‘self‐concept’, ‘prior experience’, ‘readiness to learn’, ‘orientation to learning’ and ‘motivation to learn’ (Knowles et al. 2020, 5). As nurses are adult learners who are expected to apply theoretical knowledge to clinical problem‐solving, these principles provide a relevant basis for discussing the findings in the context of adult learning and transition support in clinical practice.

## 3 Aim

This study aimed to explore how a nurse orientation programme is reflected in nurses' daily clinical experiences from the participants' perspectives and to discuss the findings in relation to Knowles' andragogy. Accordingly, the following questions were addressed:

1. How do nurses experience the orientation programme process in the institution as a whole?
2. How are the theoretical, simulation and communication/interaction components of the orientation programme perceived as being reflected in clinical practice?
3. According to nurses' views, what are the areas for improvement to strengthen the perceived clinical effectiveness and transition‐support function of the orientation programme?

## 4 Methods

The Consolidated Criteria for Reporting Qualitative Research (COREQ) checklist was used to guide the reporting of the study (Tong et al. 2007).

### 4.1 Design

**FIGURE 1:**
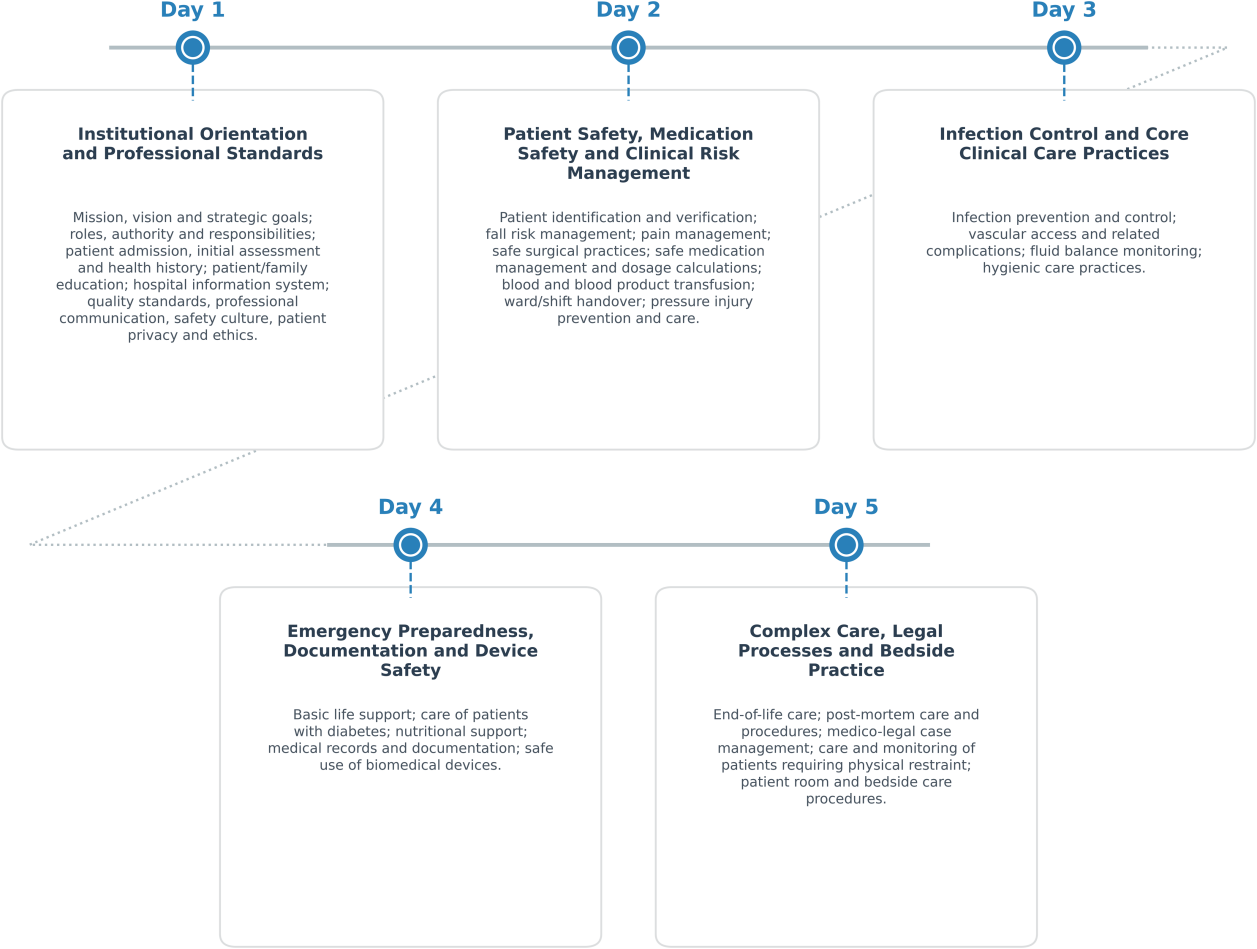
Five‐day nurse orientation programme timeline.

This study used a descriptive phenomenological design to examine how a nurse orientation programme is reflected in nurses' clinical experiences, based on participants' descriptions. Grounded in Husserl, this design aims to reveal the essential meanings and general structure of experience based on participant descriptions (Giorgi and Giorgi 2003). Consistent with this methodological orientation, descriptive phenomenology was preferred because the research aimed to describe the essential meaning of nurses' orientation experiences rather than to develop an interpretive account of lifeworld meanings, produce a general qualitative description, or examine the culture of a clinical unit. The phenomenon of interest was defined as the nurse orientation programme process and participants' perceived transfer of training outcomes into clinical practice. Details of the nurse orientation programme are presented in Figure 1.

Figure 1 presents the progression of the 5‐day nurse orientation programme based on the institution's standard orientation curriculum.

### 4.2 Theoretical Framework

Malcolm Knowles' andragogy provided a relevant theoretical perspective for discussing adult learning in the context of nurse orientation. However, andragogy was not used as an a priori coding framework during data analysis. The six principles of andragogy were not converted into predetermined codes, categories or themes and were deliberately set aside during coding and theme development to preserve the descriptive phenomenological orientation of the study. Instead, the analysis was conducted inductively, and themes were derived from participants' descriptions of their orientation experiences. After the inductive thematic structure had been developed, Knowles' principles were used in the Discussion to consider the findings in relation to adult learners' need to know, prior experience, readiness to learn, problem‐centred learning, motivation and professional autonomy (Knowles et al. 2020). Knowles' andragogy was selected for its close alignment with nurse orientation as an adult learning process involving prior experience, readiness for role‐related responsibilities, problem‐centred learning and immediate clinical application.

### 4.3 Sampling and Recruitment

The study participants were 13 nurses actively working in a private hospital in Istanbul. Criterion and maximum variation sampling were combined because purposeful sampling strategies can be used together to define a shared eligibility criterion while also capturing relevant variation within the sample (Patton 2015). Inclusion required completion of the institution's 5‐day ‘Nurse Orientation Training’ programme (Figure 1) within the previous 3 months and commencement of active clinical duty. To ensure variation, nurses with different educational backgrounds, levels of professional experience and clinical unit assignments were included. This allowed the identification of common patterns in orientation training experiences among both newly graduated and experienced nurses. Participants' demographic and professional characteristics are presented in Table 1.

**TABLE 1:** Participant information.

Participant	Gender	Age	Education level	Professional experience (month/year)	Unit
N1	Male	25	Bachelor's	4 years	Inpatient ward
N2	Female	25	Master's	2 years	Intensive care unit
N3	Female	24	Bachelor's	2 years	Intensive care unit
N4	Female	24	Bachelor's	2 years	Inpatient ward
N5	Female	27	Bachelor's	4 years	Inpatient ward
N6	Female	26	Bachelor's	4 years	Inpatient ward
N7	Female	23	Bachelor's	6 months	Inpatient ward
N8	Female	22	Bachelor's	1 year	Inpatient ward
N9	Female	27	Bachelor's	2 years	Inpatient ward
N10	Female	27	Bachelor's	2 years	Emergency department
N11	Female	23	Bachelor's	3 months	Inpatient ward
N12	Female	25	Vocational high school	3 years	Inpatient ward
N13	Female	24	Bachelor's	1 year	Inpatient ward

Table 1 shows that participants were aged 22–27, most held bachelor's degrees, and their professional experience ranged from 3 months to 4 years across inpatient, intensive care and emergency settings. The sample included four early‐career nurses with 1 year or less of experience and nine experienced nurses with more than 1 year of experience, allowing variation in orientation experiences across different levels of professional experience.

### 4.4 Data Collection

Research data were collected through semi‐structured interviews to explore participants' subjective experiences and views on the phenomenon. The interview guide, developed in line with the relevant literature and the research questions, consisted of 12 open‐ended questions. The interview guide was reviewed by two experts in nursing and educational sciences for relevance, clarity and comprehensiveness, and was finalised after linguistic and semantic revisions.

Face‐to‐face interviews were conducted between February and March 2026 at times arranged around participants' clinical work schedules. Interviews took place in a quiet, private room within the hospital. No one other than the participant and the interviewer was present. Data were collected by the third researcher, a female nurse educator with an MSc degree, employed at the institution and experienced in conducting qualitative interviews. No participant refused to participate or dropped out during the study. Although the interviewer had interacted with the participants during the orientation training, the interviewer was not the participants' administrative supervisor and did not play any role in performance evaluation or promotion processes. To reduce the potential effects of the interviewer's role within the institution, it was clearly stated to the participants before each interview that their answers would not affect their working conditions, data would be reported anonymously, and they could withdraw from the study at any time. During the interview process, the third researcher kept reflexive notes to monitor potential biases. These measures were intended to reduce social desirability pressure. Interviews were audio‐recorded to prevent data loss. A single interview session was held with each participant, and repeat interviews were not required. Interviews were conducted after the completion of the orientation training and independently of internal evaluation processes. Interviews lasted 25–35 min, depending on the depth of participants' responses.

### 4.5 Data Analysis

Data were analysed using Colaizzi's (1978) seven‐step descriptive phenomenological approach. Interviews were transcribed verbatim and read repeatedly to achieve familiarity with the data. Significant statements were identified, meanings were formulated, and these were organised into sub‐themes and main themes. The findings were then synthesised into a comprehensive description of the phenomenon and its essential structure. Participant validation was sought at the final stage (Abu Shosha 2012).

The analysis was conducted inductively and was not guided by a pre‐existing codebook. During the initial coding and theme development stages, Knowles' principles were deliberately set aside and were not used as coding categories or as a thematic template. Significant statements were first identified from the interview transcripts, meanings were formulated from participants' own descriptions, and sub‐themes and main themes were generated through repeated comparison across transcripts. Coding and theme development were carried out manually. To support bracketing, the researchers repeatedly returned to the raw data to check alignment between codes, themes and participants' accounts. Only after this inductive thematic structure had been agreed upon by the researchers were the findings discussed in relation to Knowles' andragogical principles in the Discussion. This separation was maintained to avoid imposing the theoretical framework on participants' accounts.

### 4.6 Ethical Considerations

Ethical approval for this study was obtained from the Marmara University Institute of Educational Sciences Research and Publication Ethics Committee prior to data collection (Approval date: 06 February 2026; Approval No. 02‐34). All participants provided voluntary informed consent before the interviews.

### 4.7 Rigour

To enhance credibility and scientific rigour, the strategies for qualitative rigour recommended by Merriam and Tisdell (2016) were followed. Three researchers independently coded the transcripts and then compared codes and themes until consensus was reached. Reflexive notes were kept throughout the analysis to monitor prior assumptions and potential biases, and coding decisions and analytic steps were documented. Data collection continued until saturation was reached; saturation became evident around the tenth interview, and three additional interviews were conducted to confirm it. To support transferability, the research context, sample and process were described in detail. Direct quotations were included in the findings section, and participants were coded as N1, N2, and so on to preserve anonymity. An audit trail documenting all stages of the research was retained to support consistency and confirmability.

## 5 Findings

**FIGURE 2:**
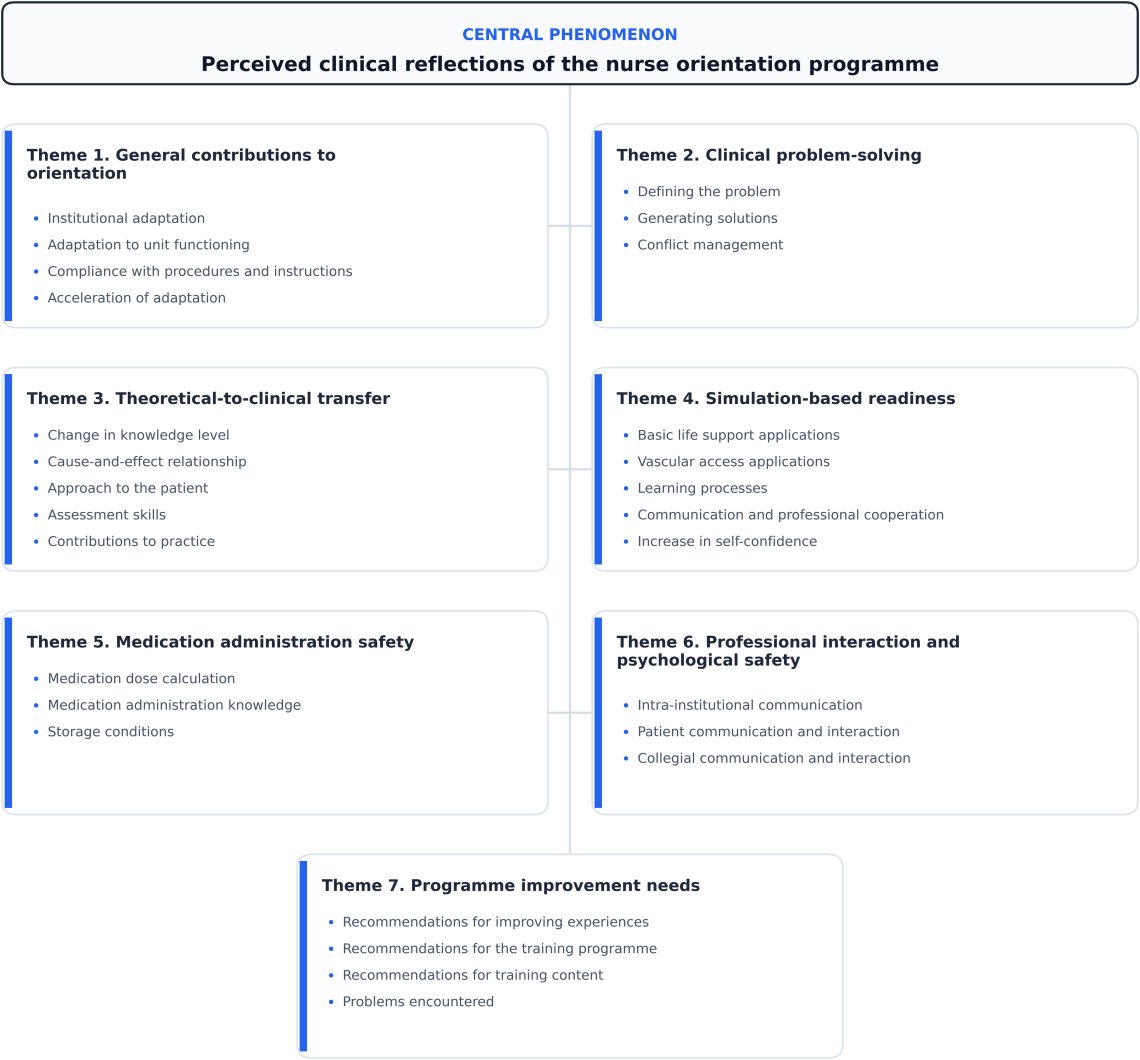
Thematic structure of nurses' perceived clinical reflections of the orientation programme.

Seven main themes and 27 sub‐themes were identified regarding how participants perceived nurse orientation training as being reflected in clinical practice. The thematic structure is presented in Figure 2, and an example of the analysis flow is shown in Table 2.

**TABLE 2:** Example of Colaizzi analysis.

Colaizzi analysis steps	Content
Significant statement	‘It was good for our process of getting to know the hospital. We met our managers; we met our education nurses. We learned the mission and vision of the institution. Coming to our unit knowing the rules allowed us to work more comfortably. It accelerated our adaptation process (N6)’
Formulated meanings	The participant perceived orientation training as reducing initial uncertainty and strengthening the sense of belonging by making the institution's structure and key actors visible Learning the rules before starting in the unit was described as facilitating adaptation by supporting more comfortable and controlled working in the clinical environment
Relevant theme/sub‐theme	Theme 1—General contributions to orientation; Sub‐theme 1.1—Institutional adaptation

Figure 2 presents the central phenomenon, main themes and sub‐themes regarding nurses' perceived clinical reflections of the orientation programme.

### 5.1 Theme 1. General Contributions to Orientation

Participants perceived orientation training as accelerating organisational integration and reducing clinical uncertainty.

- Sub‐theme 1.1. Institutional adaptation (*focus points: getting to know the institution, learning mission and vision, meeting managers*): Participants stated that recognising the structure of the institution and its key actors was perceived as reducing initial anxiety and strengthening the sense of belonging (N6, N11).

Since I met the education nurses and my managers, I started feeling more comfortable. (N11)

- Sub‐theme 1.2. Adaptation to unit functioning (*focus points: working hours, unit rules, daily workflow*): Participants described understanding the order and functioning of the unit beforehand as supporting their perceived confidence and sense of control in the clinic (N2, N3, N4, N5, N6, N7, N8).

I learned my unit better. Since I learned the working hours, rules and unit functioning, I felt more self‐confident in the unit. (N4)

- Sub‐theme 1.3. Compliance with procedures and instructions (*focus points: medication administration instructions, medical device usage, document usage, infection precautions*): Participants perceived that learning standard processes supported their ability to follow safety‐related practices and increased their awareness of potential clinical errors (N1, N5, N7, N8, N11).

They really explained everything we needed to learn in detail; from heat and humidity control to the crash cart, form usage, to medication administration… When I returned, I had a command of everything. I performed my practices without stumbling. (N8)

- Sub‐theme 1.4. Acceleration of adaptation (*focus points: prior knowledge and readiness, facilitating transition to the clinic, shortening adaptation time*): Participants stated that the preparation acquired before starting in the unit was perceived as facilitating the transition to the clinic and shortening the adaptation period (N2, N3, N6, N10, N11, N12).

Knowing certain information beforehand allowed me to respond to patients more easily. They had introduced the room before. For example, when there was a problem with the ventilation, I was able to respond to this problem faster. (N12)

### 5.2 Theme 2. Clinical Problem‐Solving

Participants perceived orientation training as guiding them in defining problems, generating solutions and using institutional processes in the clinic.

- Sub‐theme 2.1. Defining the problem (*focus points: understanding clinical problems, job description and area of responsibility*): Participants felt that the training supported faster recognition of ‘what the problem is’ in clinical situations and clarification of responsibility boundaries (N1, N4, N5, N10).

Trainings contribute more to interpreting problems. Compared to a nurse who has no perspective on issues such as what applications can be done to a postoperative patient or how nursing care is applied, a nurse who receives theoretical training has more advantages. When I experienced a problem, the training slides came to my mind due to visual memory. (N1)

I learned what my own responsibilities were and what the physician's responsibilities were. For example, I learned that obtaining consent from the patient was not my duty. When I encounter such a request, I can express myself more clearly and avoid taking responsibility outside my role. (N4)

- Sub‐theme 2.2. Generating solutions (*focus points: using learned knowledge in practice, access to procedures/documents, error management*): Participants associated knowing the path to follow in possible errors with a perceived ability to act based on evidence and to proceed more systematically in the solution‐generation process (N1, N2, N3, N4, N7, N8, N10, N11, N12).

Since I was a new graduate anyway, I hadn't experienced many things practically… I didn't know how to administer which drug, what kind of side effects it had, or how I should intervene. Medication administrations were the thing I struggled with the most at first. The training allowed me to find solutions to these. (N3)

- Sub‐theme 2.3. Conflict management (*focus points: anger management, preventing communication problems*): Participants reported feeling better able to maintain emotional control in challenging interactions, preserve professional communication and manage potential conflict (N6, N9, N13).

I am normally an aggressive person. Thanks to this training programme, it taught me that I need to maintain my calm in the face of difficulties. It improved my communication. (N6)

### 5.3 Theme 3. Theoretical‐To‐Clinical Transfer

Participants described theoretical training as being reflected in clinical experience through perceived changes in knowledge level, theory‐practice connection, approach to the patient and clinical assessment skills.

- Sub‐theme 3.1. Change in knowledge level (*focus points: knowledge acquisition, updating knowledge, acceleration of learning*): Participants reported that theoretical training helped them refresh existing knowledge, acquire new knowledge, and feel better prepared to begin clinical practice (N1, N3, N6, N7).

Since you have taken the theoretical training before starting to work directly in the unit, you perform applications on the patient knowing something in the clinic. You don't turn into a fish out of water. It was very good to establish the foundation and start working. I also updated myself. (N6)

- Sub‐theme 3.2. Cause‐and‐effect relationship (*focus point: theory‐practice connection*): Participants stated that being able to generate answers to the question ‘why’ supported their perceived clinical decision‐making processes (N3, N6, N8, N10).

The information we received contributed to my understanding of what we do and why in practice and to learning faster. (N3)

You cannot apply something if you do not know its theory. When we listened to the theoretical part, we learned how to apply it. Practice makes it more permanent. When you apply something in the clinic after learning its theory, it becomes more permanent. You learn what you are doing, why you are doing it, and how to do it. (N8)

- Sub‐theme 3.3. Approach to the patient (*focus points: developing perspective, patient safety, patient education*): Participants expressed that the training supported a more standardised approach to the patient and contributed to their perceived safety‐related preparedness (N2, N7, N11).

I applied the trainings I received theoretically beforehand much more comfortably and confidently while applying them. I learned what we do and why. I approached the patient knowing these. I was able to give clearer answers to the questions they asked. (N11)

- Sub‐theme 3.4. Assessment skills (*focus points: post‐application monitoring, symptom and general condition assessment*): Participants noted that the training supported their perceived competence in holistic patient assessment, symptom tracking, and post‐application monitoring steps (N4, N5, N6, N12, N13).

…After giving an oral drug or administering a drug intravenously or intramuscularly, I learned the onset times, how often I would make assessments, and what I should pay attention to along with their pain… in the training. This contributed to my application and assessment. (N4)

Pain assessment forms are explained clearly in the training, including how often pain should be assessed. If these had not been explained before we came to the ward, we might have had difficulty. We question the patient until the pain reaches the desired level and record it with the vital signs. If this had not been explained in the training, pain assessment might not have been performed properly. Because it was covered in the training, we do not experience any disruption in this process. (N5)

- Sub‐theme 3.5. Contributions to practice (*focus points: practicality, speed, time management*): Participants stated that theoretical training contributed to their perceived ability to conduct clinical applications more fluidly, in a more planned manner, and more effectively in terms of time management (N2, N9).

All the information explained taught us how our perspective towards patients should be within the hospital. It allowed us to perform the applications we do to the patient quickly. (N2)

### 5.4 Theme 4. Simulation‐Based Readiness

Participants described simulation training as strengthening their perceived readiness for emergency management and basic life support, familiarity with vascular access applications, retention of learning, intra‐team cooperation and self‐confidence.

- Sub‐theme 4.1. Basic life support applications (*focus points: composure, code blue, early intervention, equipment‐material management*): Participants stated that simulation increased their perceived readiness in emergency management and helped them feel more able to apply the correct steps in critical moments (N2, N3, N4, N5, N6, N7, N8, N9, N10, N11, N12, N13).

I have never encountered CPR so far. Therefore, I do not fully trust myself yet, but at least theoretically I know what I should and should not do. I learned how to call a code blue, where the defibrillator is, and how it should be checked. (N7)

Triage, giving a code blue call, and intervention steps to be done until the team arrives allowed me to do them quickly. (N10)

- Sub‐theme 4.2. Vascular access applications (*focus points: peripheral venous catheter insertion skills, catheter care, port needle, advanced vascular access devices*): Participants expressed that recognising the tools and learning the care/application steps supported their perceived safety‐related preparedness in clinical practice (N1, N2, N3, N4, N5, N6, N7, N8, N9, N10, N11, N13).

When intravenous access was demonstrated step by step, and waste separation was practised, it was very important. No matter how much you know, you can still panic. When you first practise on the model and learn basic information such as the angle of needle insertion, when to withdraw it, and where to apply pressure on the catheter, you feel more confident at the bedside and your panic decreases. (N8)

… the applied port needle insertion and removal trainings I received provided great support while performing applications on the patient. We had heard the sound the needle makes while trying it in the training. When I heard that sound while inserting it in the clinic, I was sure I inserted it correctly. It allowed me to feel more confident in my practice. (N10)

- Sub‐theme 4.3. Learning processes (*focus points: retention, enjoyable learning, realism*): Participants perceived simulation as reinforcing learning, providing opportunities to practise before patient care, and making information more memorable (N1, N5, N6, N7, N9, N11, N13).

Simulation trainings were very useful in terms of both retention and being closer to real situations. I was able to respond faster to problems when I encountered real situations. (N13)

- Sub‐theme 4.4. Communication and professional cooperation (*focus points: intra‐team communication, teamwork, role sharing*): Participants expressed that coordination and joint decision‐making processes in scenario‐based applications strengthened their perceived clinical cooperation (N9, N10, N11).

We applied all steps as a team on the mannequin just like a real event. It both taught teamwork and ensured the application was done correctly. (N11)

- Sub‐theme 4.5. Increase in self‐confidence (*focus point: confidence in transition to clinical practice*): Participants reported feeling more confident during the transition to practice, particularly in interventions that initially caused anxiety (N2, N4, N8, N10, N13).

When I came to the clinic, I performed how to clamp, how to dress, and how to remove the catheter in a patient with a central venous catheter very easily. … It increased my confidence in myself. (N4)

### 5.5 Theme 5. Medication Administration Safety

Participants described orientation training as supporting their perceived preparedness and confidence in medication administration.

- Sub‐theme 5.1. Medication dose calculation (*focus points: dose calculation confidence and awareness of calculation‐error risk*): Participants associated this training with greater confidence in dose calculation and a perceived reduction in error risk (N1, N4, N5, N6, N7, N13).

The medication dose calculations we saw in training allowed me to prepare medication more accurately in the field. Dose calculations are especially very important in paediatric patients…. (N1)

- Sub‐theme 5.2. Medication administration knowledge (*focus points: specific drugs, routes of administration, administration steps, solution compatibility*): Participants associated this training with a greater perceived ability to follow standards in medication preparation and administration processes, anticipate potential complications, and feel more confident about medication administration safety (N2, N3, N8, N9, N10, N11, N12, N13).

Some antibiotics have special dilution conditions; there are interacting drugs. We learned these. These pieces of information come to our minds more and become permanent while administering drugs, especially right after the training. Then the information you learned settles. (N8)

- Sub‐theme 5.3. Storage conditions (*focus points: appropriate temperature/light conditions; shelf life, pre‐application check*): Participants emphasised the importance of appropriate medication storage conditions and pre‐administration checks (N4, N7, N8, N13).

We learned the storage conditions of drugs. If a drug needs to come via cold chain, I check this when the drug reaches me. If it is not suitable, I send it back. This prevented me from administering an inappropriately stored drug. (N7)

### 5.6 Theme 6. Professional Interaction and Psychological Safety

Participants described communication and interaction as supporting their perceived clinical functioning.

- Sub‐theme 6.1. Intra‐institutional communication and interaction (*focus points: inter‐unit communication, service/shift handovers*): Participants associated this with stronger perceived coordination, recognition of communication channels, and more systematic execution of handover processes (N1, N2, N3, N8, N13).

When you experience any trouble, when you have a question, we talk on the phone, we meet, we stay in communication with every floor… We learn if there is a different process for patient care on another floor of the hospital. Because different physicians have different expectations. That's why it is very good for communication. (N1)

- Sub‐theme 6.2. Communication and interaction with the patient (*focus points: correct communication, explaining the process, trust relationship*): Participants perceived the training as supporting their ability to communicate appropriately with patients, explain care processes and establish trust (N5, N9).

We came to the ward already knowing the clinic. The training provided a major contribution, especially in terms of communication. At least, we received training on how to communicate with patients and how medication treatments should be administered. All of these contributed to the process. (N5)

- Sub‐theme 6.3. Communication and interaction with colleagues (*focus points: meeting, trust, experience sharing, cooperation*): Participants expressed that increased interaction with nurses from different units strengthened perceived solidarity and facilitated cooperation in the clinic (N1, N2, N4, N5, N6, N7, N8, N9, N11, N12, N13).

You meet people. Most nurses start after school; I started like that too. Since it was a school environment, we mingled faster. It provided a softer transition from school to work life. (N1)

When you know people in the place where you work, when you can drink tea or coffee together during breaks, and when you can consult someone when something happens, it makes you feel that you are in a more peaceful and safer environment. (N6)

### 5.7 Theme 7. Programme Improvement Needs

Participants presented recommendations for the development of the programme and indicated areas for strengthening regarding training content and method. Furthermore, some participants voiced logistical difficulties.

- Sub‐theme 7.1. Recommendations for improving experiences (*focus points: scientific studies, participation in trainings, learning from observation and experience*): Participants emphasised continuing professional development, access to evidence‐based knowledge and learning from experienced staff (N2, N4, N6, N7, N9, N10, N11, N12, N13).

After starting work, nurses should work with different nurses in the clinic. Everyone may have different techniques and shortcuts. Therefore, they should work with different people, benefit from everyone's experience, continuously discuss unknown issues with the charge nurse, review the literature, participate in training programmes, and avoid working by rote. (N7)

- Sub‐theme 7.2. Recommendations for the training programme (*focus points: in‐unit training, increasing simulation, interactive methods, social sharing, rotation, training repetition*): Participants expressed expectations regarding the delivery and continuity of the programme (N1, N2, N3, N5, N6, N7, N8, N9, N10, N11, N13).

When returning to the field after orientation training, there should be specialised in‐unit training. In‐unit case‐based training programmes should be increased. (N1)

A student‐like environment was created during the training. I felt this because of the setting. It was like a classroom environment. Beforehand, we could have had coffee together with the educators as colleagues, or there could have been an introductory breakfast. I would have preferred a colleague relationship rather than a teacher–student relationship. (N7)

- Sub‐theme 7.3. Recommendations for training content (*focus point: hygienic care*): One participant suggested placing greater emphasis on hygienic care (N1), whereas the other participants reported no significant deficiencies in the programme content (N2, N3, N4, N5, N6, N7, N8, N9, N10, N11, N12, N13).

As education nurses, you talk about every subject. There is no subject I think is missing. Hygienic care can be emphasised. We frequently have care patients in our hospital. (N1)

- Sub‐theme 7.4. Problems encountered (*focus points: transportation difficulty, long training hours*): Most participants stated they did not experience significant difficulties in the process (N1, N2, N3, N5, N9, N12, N13), while a limited number of participants emphasised problems stemming from logistics and time management, such as transportation difficulties (N6, N7, N8) and long training hours (N4, N10).

The hours of the training were long. It was difficult to stay alert in the afternoon, especially after lunch. (N4)

I only experienced difficulty in transportation …. (N6)

The essence of the phenomenon was that participants perceived nurse orientation training as reducing uncertainty and supporting their perceived safety‐related preparedness and confidence during the transition into clinical practice by linking theoretical grounding, simulation, medication safety and communication support during the early period of employment.

## 6 Discussion

The central phenomenon emerging from the findings was that nurses perceived the orientation programme as a transition‐support mechanism that reduced uncertainty and supported their transition into clinical practice. Rather than reflecting a single outcome, this transition‐support function was described through interconnected experiences of organisational adaptation, clinical problem‐solving, theory‐practice transfer, simulation‐based readiness, medication administration safety, professional interaction and programme improvement needs. From this perspective, the orientation programme was not perceived merely as an introductory institutional activity, but as a structured process through which nurses made sense of their new responsibilities, developed confidence and connected prior and newly acquired knowledge with everyday clinical demands.

### 6.1 Orientation as a Transition‐Support Mechanism

Participants' accounts suggest that orientation was perceived to support the transition into practice by making institutional expectations, unit functioning, clinical procedures and available support systems more visible before nurses entered full clinical responsibility. One participant stated, ‘We learned the mission and vision of the institution… It accelerated our adaptation process’ (N6). Another participant explained that orientation supported problem‐solving in practice: ‘…I find solutions to my problems by looking at the relevant documents’ (N2). These accounts suggest that orientation was perceived not only as an introductory process, but also as a practical support mechanism that nurses could draw on after entering clinical practice. This finding is consistent with previous studies showing that structured orientation and transition‐support programmes can facilitate adaptation, confidence and professional integration among newly hired nurses (Ernawaty et al. 2024; du Toit et al. 2026). However, the present study adds to this literature by showing how nurses perceived different components of the orientation programme as being reflected in everyday clinical situations after programme completion.

The findings indicate that transition support was not limited to organisational adaptation. Participants also described orientation as helping them define clinical problems, access procedures and documents, clarify role boundaries and generate solutions in practice. Theoretical training was perceived as meaningful when it helped nurses understand what they were doing, why they were doing it, and how they should act in clinical situations. As one participant stated, ‘The information we received contributed to my understanding of what we do and why in practice’ (N3). This suggests that theoretical knowledge becomes meaningful when it is clearly connected to practice. Similarly, simulation training appeared to support perceived readiness by allowing participants to rehearse emergency responses, vascular access procedures, teamwork and psychomotor skills before encountering similar situations in patient care. As one participant explained, ‘We had heard the sound… while trying it in training. When I heard that sound… in the clinic, I was sure I inserted it correctly’ (N10). This illustrates how prior experiential learning may support perceived confidence and preparedness in real clinical settings. This is consistent with recent literature suggesting that simulation‐based training plans may contribute to patient safety‐related outcomes (Muñoz et al. 2025).

Medication administration safety emerged as a particularly salient part of this transition‐support process. Participants described dose calculation, medication preparation, administration protocols and storage conditions as areas that supported perceived confidence and safety‐related preparedness. This finding is important because medication administration is closely linked to professional responsibility, fear of error and patient safety. In this respect, patient safety appeared to function as a strong internal motivator for learning. At the same time, literature also highlights that fear of blame or punishment may affect medication error reporting (Yang et al. 2025). Therefore, orientation programmes may need to combine medication safety content with messages that promote psychological safety and a learning‐oriented culture (Hendy et al. 2025).

Professional interaction and psychological safety also appeared central to participants' transition experiences. Knowing other nurses, being able to consult colleagues and communicating with staff from different units were perceived as factors that strengthened trust and reduced anxiety. One participant stated that ‘…being able to consult… makes a person feel… peaceful and safe’ (N6). This finding is consistent with research highlighting the role of peer support, mentorship, and emotional support in the well‐being, job satisfaction and retention of nurses during transition (Raqueno et al. 2025; du Toit et al. 2026). Thus, orientation may be understood as both a knowledge‐transfer process and a relational process that supports belonging, confidence and professional identity formation. To consider the learning dynamics underlying this transition‐support mechanism, the findings were subsequently discussed in relation to Knowles' andragogical principles.

### 6.2 The Transition‐Support Process in Relation to Andragogy

After the inductive analysis was completed, the seven themes were discussed in relation to Knowles' principles of andragogy (Knowles et al. 2020). Table 3 should therefore be read as a post hoc matrix rather than as evidence of a deductive or framework‐driven coding process. The apparent links between the themes and andragogical principles were considered during the post‐analysis discussion, not during initial code generation or theme development.

**TABLE 3:** Post hoc matrix relating inductively derived themes to Knowles' andragogical principles.

Inductively derived theme	The learner's need to know	Self‐concept of the learner	Prior experience of the learner	Readiness to learn	Orientation to learning	Motivation to learn
General contributions to orientation	✓					
Clinical problem‐solving					✓	
Theoretical‐to‐clinical transfer				✓		
Simulation‐based readiness			✓			
Medication administration safety						✓
Professional interaction and psychological safety		✓				
Programme improvement needs		✓				

*Note:* This post hoc matrix was developed after the inductive analysis to discuss the findings in relation to Knowles' andragogical principles; checkmarks do not indicate a priori codes or predetermined categories.

Knowles' andragogy was used not to validate or structure the seven themes, but to discuss how the perceived transition‐support function of orientation related to key dimensions of adult learning. Participants' need to know was evident in their emphasis on understanding institutional expectations, clinical procedures and the reasons behind practice requirements. Their prior experience shaped how they understood and responded to simulation, medication safety and clinical scenarios. Readiness to learn was reflected in the way orientation became meaningful when it was connected to immediate professional responsibilities. A problem‐centred orientation to learning was evident in participants' descriptions of using documents, procedures and training content to solve clinical problems. Motivation to learn was closely linked to patient safety, professional responsibility and the desire to feel competent in practice. Finally, self‐concept was reflected in participants' need for autonomy, collegial communication and confidence during the transition into clinical work.

Nevertheless, Knowles' framework was used cautiously in this study. Earlier critiques questioned whether andragogy should be understood as a distinct and unified theory of adult learning or rather as a philosophical position and set of assumptions about adult education (Hartree 1984; Pratt 1993). More recent scholarship also describes andragogy as a foundational and practical contribution to understanding adult learners, while acknowledging that it has been subject to substantial critique over the last five decades (St. Clair 2024). Therefore, Knowles' principles were used here as a sensitising theoretical perspective for discussing the findings rather than as a definitive explanatory framework.

### 6.3 Programme Continuity and Areas for Improvement

Although participants' accounts were predominantly positive, their recommendations suggest that orientation should not be viewed as a one‐time event. Suggestions related to in‐unit training, increased simulation, rotation, training repetition, social sharing and continuing support indicate that nurses perceived transition as an ongoing process. Difficulties related to training duration and access to the training locations also show that programme design should balance institutional requirements with adult learners' practical needs and autonomy. Accordingly, orientation may be better conceptualised as a continuing transition‐support structure that extends beyond initial institutional introduction and supports nurses' adaptation within the clinical field.

### 6.4 Strengths and Limitations of the Work

A strength of this study lies in its phenomenological focus on how orientation is perceived as being reflected in everyday clinical practice through nurses' own accounts. However, the study was conducted with a small sample of nurses from a single private hospital in Istanbul; therefore, the findings should be considered within this specific institutional context. Since the data were based on self‐reported interviews conducted within 3 months of orientation training, the findings may have been influenced by recall bias and social desirability. Although several steps were taken to reduce this risk, the interviewer's dual role as an education nurse involved in the orientation programme may have influenced participants' willingness to express critical views. The predominantly positive nature of participants' accounts should therefore be interpreted cautiously; it is possible that the orientation programme was genuinely perceived as supportive, but it is also possible that the interviewer's institutional role limited the extent to which participants felt comfortable expressing criticism. In addition, the study reflects only nurses' perspectives and does not include the views of managers, educators, or other stakeholders. Accordingly, the findings are intended to provide contextual and transferable insights rather than broad generalisations.

### 6.5 Implications for Practice

Given the single‐site qualitative design and the small sample size, the following implications should be interpreted as tentative practice considerations rather than generalisable policy directives. The findings suggest that healthcare institutions with similar orientation structures may consider strengthening case‐based, problem‐centred and simulation‐supported learning opportunities within nurse orientation programmes. In‐unit reinforcement, mentorship and access to protocols and clinical documents may also be useful in supporting nurses after the formal orientation period. The participants' accounts further suggest that medication safety content may warrant continued emphasis, particularly in relation to dose calculation, drug compatibility, administration procedures and storage conditions.

Training environments that promote peer interaction, access to collegial consultation and psychological safety may also support nurses' perceived confidence during transition into clinical practice. In addition, a non‐punitive and learning‐oriented safety culture may be relevant when addressing clinical errors within orientation and transition‐support processes. The findings also indicate that orientation may be better conceptualised as a continuing transition‐support process rather than as a one‐time introductory intervention. Orientation and transition‐support programmes may represent a potential institutional investment in perceived clinical preparedness and safety‐related awareness; possible effects on care quality and staff retention require further examination in larger, multi‐site studies. Rather than offering definitive policy recommendations, this study provides context‐specific insights that may inform the refinement of orientation and transition‐support programmes.

### 6.6 Recommendations for Future Research

Future studies should examine the long‐term effects of nurse orientation programmes on clinical performance, patient safety outcomes, nurse retention and professional adaptation. Comparative studies across different institutional settings may help clarify how organisational context shapes orientation experiences and outcomes. In addition, future research may benefit from using multi‐source data, such as direct observation, educator and manager perspectives, documentation audits and clinical outcome indicators, to examine whether nurses' self‐reported perceptions correspond with observable changes in clinical practice.

## 7 Conclusion

This descriptive phenomenological study suggests that nurses perceived the orientation programme as facilitating organisational integration, supporting clinical problem‐solving, and contributing to their perceived safety‐related preparedness and confidence during the transition into clinical practice. Participants described the perceived transfer of theoretical learning into clinical work through experiential learning opportunities, simulation, medication safety content and practice‐oriented support. Discussed in relation to Knowles' andragogical principles, the findings highlight the perceived value of orientation processes that are responsive to adult learners' needs for relevance, experience‐based learning, problem‐centred support and professional autonomy. More broadly, the study contributes to conceptualising nurse orientation not merely as an introductory institutional requirement, but as a continuing transition‐support process through which adult learners make sense of new professional responsibilities. However, because the findings are based on nurses' self‐reported accounts, further studies using observational, multi‐source and outcome‐based designs are needed to examine whether these perceived benefits correspond with measurable changes in clinical practice, patient safety and professional adaptation.

## Author Contributions

**Cansu Çavuşoğlu:** investigation, writing – original draft, validation, methodology, visualization, formal analysis, data curation, conceptualization. **Semih Çayak:** conceptualization, methodology, data curation, formal analysis, validation, writing – review and editing, writing – original draft, visualization. **Sibel Öztürk Demir:** methodology, validation, visualization, writing – review and editing, writing – original draft, conceptualization, formal analysis, data curation.

## Funding

The authors have nothing to report.

## Disclosure

Published accounts: This work has not been previously presented, published as an abstract, posted as a pre‐print, or included in a thesis.

## Ethics Statement

Ethical approval for this study was obtained from the Marmara University Institute of Educational Sciences Research and Publication Ethics Committee prior to data collection (Approval Date: 06.02.2026; Approval No.: 02‐34).

## Conflicts of Interest

The authors declare no conflicts of interest.

## Data Availability

The data that support the findings of this study are available on request from the corresponding author. The data are not publicly available due to privacy or ethical restrictions.
